# Exploring Factors Associated With Professional Identity Among Midwifery Students in Tehran Following the COVID‐19 Crisis: A Cross‐Sectional Study

**DOI:** 10.1002/hsr2.72009

**Published:** 2026-04-11

**Authors:** Sahar Borji‐Navan, Homa Sadeghi Avval Shahr, Shima Haghani, Sanam Borji‐Navan

**Affiliations:** ^1^ Student Research Committee, School of Nursing and Midwifery Iran University of Medical Sciences Tehran Iran; ^2^ Department of Midwifery, Faculty of Nursing and Midwifery Iran University of Medical Sciences Tehran Iran; ^3^ Department of Biostatistics, Nursing and Midwifery Care Research Center Iran University of Medical Sciences Tehran Iran; ^4^ Student Research Committee, School of Nursing and Midwifery Shahroud University of Medical Sciences Shahroud Iran

**Keywords:** COVID‐19, cross‐sectional, education, midwifery students, pandemic, professional identity

## Abstract

**Background and Aims:**

The COVID‐19 pandemic has significantly impacted healthcare education and professional development. This study aimed to determine the predictors of professional identity among midwifery students in Tehran in the transition from the COVID‐19 pandemic crisis.

**Methods:**

A cross‐sectional study was conducted using a convenience sampling method to include 140 undergraduate midwifery students from three public medical universities in Tehran. Data were collected using a demographic questionnaire, the Professional Identity in Nursing Students Questionnaire (PINSQ), and a Visual Analog Scale (VAS) to assess the impact of the pandemic on professional identity.

**RESULTS:**

The mean score for professional identity was 76.22 (SD = 9.44). Multiple linear regression results indicated that ethnicity was a significant predictor, with students from other ethnic groups reporting a higher professional identity score than Fars students (β = 4.192, *p* < 0.05). In contrast, age was a significant negative predictor of professional identity (β = −0.979, *p* < 0.05). Additionally, third‐year students had significantly lower professional identity scores compared to graduates (β = −5.178, *p* < 0.05). Furthermore, professional identity was positively associated with higher parental education levels and a positive perception of the pandemic′s impact on the social image of the profession. Conversely, a significant negative correlation was found between professional identity and the perceived levels of informational and emotional support from professors.

**Conclusions:**

This study reveals that professional identity in midwifery students is significantly shaped by demographic and educational factors. Specifically, students from minority ethnic groups and those in later academic years demonstrated stronger professional identity, while age was a negative predictor. Notably, while a positive perception of the pandemic′s impact was associated with stronger identity, perceived support from professors was paradoxically linked to weaker identity. These findings underscore the need for midwifery programs to prioritize tailored strategies that address the diverse needs of students. This includes enhancing clinical experiences, re‐evaluating the nature of faculty support, and fostering a sense of belonging to strengthen the professional identity of the future midwifery workforce.

**Ethical Code:**

IR. IUMS. REC.1401.878.

## 1 Introduction

Professional identity, a cornerstone of career development and professionalism in medical education, refers to the process of internalizing the values, beliefs, and norms of a profession to shape a professional self‐concept [1, 2, 3]. This identity is not formed in isolation; it is developed through individual experiences, social interactions, and participation within a community of practice [4, 5, 6, 7, 8, 9], allowing individuals to confidently assume their professional roles [10]. A positive professional identity is crucial, as it fosters confidence, a sense of belonging [11], job satisfaction, and career longevity [12, 13]. Furthermore, it is linked to improved patient care [14], mitigated stress [15], reduced burnout [16, 17], and the protection of the profession′s legitimacy [8, 18].

The formation of this identity typically occurs during formal education [19] and is shaped by a variety of social, personal, and communicative factors [20]. It is a primarily experiential process, involving direct interactions with patients and healthcare teams that allow students to internalize professional norms and information. Because this process is so fundamentally experiential, it is particularly vulnerable to large‐scale disruptions that alter or limit these crucial interactions [21, 22, 23].

The COVID‐19 pandemic represents one of the most significant such disruptions in modern history [10]. The resulting school closures and reduced clinical access [24] forced abrupt curriculum changes [25, 26, 27] and raised significant concerns about the impact on students′ professional identity development [28]. Historical precedent from past outbreaks like SARS showed similar effects on career perceptions [27, 29] and created ethical dilemmas during clinical experiences [30, 31]. While some students found volunteer work beneficial [4], the intense media portrayal of healthcare workers [32, 33] and pandemic‐related stressors [34] created a complex environment for professional identity formation.

For a midwife, this professional identity is forged during their student years [35, 36]. Clinical encounters, academic hurdles, and hands‐on training are the foundational elements that build not only their professional character but also their resilience and capacity for empathetic care [35, 36, 37, 38, 39, 40]. Therefore, a strong professional identity is essential for effective midwifery practice [41]. The COVID‐19 pandemic created a dual disruption for midwifery students, simultaneously moving their academic learning online while limiting vital, hands‐on clinical engagement [42]. This shift occurred within a healthcare atmosphere charged with heightened stress and personal risk, creating a uniquely challenging environment that could profoundly influence how these students developed their professional identities [43, 44, 45, 46].

Understanding how the pandemic influenced the emerging professional identity of midwifery students is critical for building more responsive and supportive educational systems [43, 44]. Despite these challenges, students are still expected to meet global midwifery standards [47]. While the pandemic′s impact on the professional identity of established practitioners is well‐studied [48, 49, 50], it remains critically underexplored among midwifery students. Therefore, this research aimed to identify the predictors of professional identity among Iranian midwifery students during the COVID‐19 pandemic to inform targeted educational interventions.

## 2 Methods

This cross‐sectional study was reported in accordance with the Strengthening the Reporting of Observational Studies in Epidemiology (STROBE) statement [51, 52].

### 2.1 Ethical Considerations

The study protocol was approved by the Ethics Committee of the Iran University of Medical Sciences (Ethics Code: IR. IUMS. REC.1401.878). All participants were explicitly informed in the study information sheet that their participation was completely voluntary and that they had the right to withdraw from the study at any time, for any reason, without any negative consequences to their academic standing or otherwise.

Furthermore, to ensure confidentiality, all questionnaires were anonymized, and no personally identifiable information was collected. The data were coded and stored on a password‐protected computer accessible only to the primary research team.

### 2.2 Study design, Setting, and participants

This study utilized a cross‐sectional, descriptive‐analytical design to explore factors associated with professional identity among midwifery students in Tehran following the COVID‐19 crisis. The research was conducted at three public medical universities in Tehran: Iran University of Medical Sciences, Shahid Beheshti University of Medical Sciences, and Tehran University of Medical Sciences.

1. Enrolled in the midwifery program in the academic year 2020‐2021 or earlier.
2. Experienced at least one academic semester during the COVID‐19 pandemic.
3. Self‐reported no cognitive or behavioral disorders.
4. Self‐reported no use of antidepressants or sedatives.
5. Self‐reported no debilitating underlying medical conditions.
6. Provided complete and valid questionnaire responses.

Students with guest or transfer status and those lacking internet access were excluded.

Our study employed a hybrid data collection model. While some participants completed the questionnaire in person, internet access was a necessary prerequisite for those who could not be reached on campus. This approach was the only feasible method to include students who were off‐site or learning remotely during the data collection period.

Data collection was conducted between March 2023 and March 2025.

### 2.3 Sampling Method and Sample Size

The study initially aimed to employ a census sampling approach, targeting the entire population of midwifery students in the participating universities. Based on official enrollment records, the total eligible population was identified as 314 students, which became the target sample size. Out of the 314 eligible students invited to participate, 140 students returned fully completed questionnaires, yielding a response rate of 44.6%.

Due to the voluntary nature of participation and the incomplete response from the total population, the final achieved sample is more accurately characterized as a non‐probability convenience sample. This is because it comprises the individuals who were accessible and willing to take part, rather than the entire intended census population.

### 2.4 Recruitment

Following approval of the study protocol by the Ethics Committee of the Iran University of Medical Sciences and obtaining necessary permissions, researchers contacted the heads of the midwifery departments at each university. A hybrid recruitment strategy was then employed.

For in‐person recruitment, Class schedules and student contact information were obtained. Researchers then attended classes to introduce the study, explain its purpose, and invite students to participate. Written informed consent was obtained from all participating students. For remote recruitment, which targeted senior students, recent graduates, contact information was obtained from the department, and they were invited to participate via phone calls and through virtual class groups. Online informed consent was obtained by requiring participants to check a mandatory confirmation box before they could access the questionnaire.

To maximize participation, follow‐up reminders were sent to non‐respondents twice a month for a period of 6 months via text messages.

### 2.5 Data Collection Tools

Three instruments were utilized for data collection in this study.

#### 2.5.1 Demographic and COVID‐19 Related Information Questionnaire

This questionnaire, developed for this study, consisted of five sections: demographic characteristics (9 questions), academic characteristics (7 questions), socio‐economic characteristics (13 questions), and information related to the COVID‐19 pandemic experience (16 questions). The questionnaire was reviewed and approved for content and face validity by five faculty members.

#### 2.5.2 Professional Identity in Nursing Students Questionnaire (PINSQ)

This 17‐item questionnaire, developed by Yu Fang Hao (2014) and adapted for midwifery students by replacing “nurse” with “midwife,” assesses professional identity across five dimensions: professional self‐image, benefit of retention and risk of turnover, social comparison and self‐reflection, independence of career choice, and social modeling. Items are scored on a five‐point Likert scale (1 = strongly disagree to 5 = strongly agree), with higher scores indicating stronger professional identity. Item 15 is reverse‐scored. The original PINSQ demonstrated a Cronbach′s alpha of 0.827, a split‐half reliability of 0.842, and good construct validity [53]. To ensure content and face validity, the questionnaire was reviewed by a panel of five faculty members from the midwifery department. Following their feedback, for reliability, internal consistency was assessed using Cronbach′s alpha on data from the final sample. The PINSQ demonstrated excellent reliability, with a Cronbach′s alpha of 0.93 for all items and values ranging from 0.42 to 0.90 for the various subscales (Table 1). Test‐retest reliability was not assessed in this study.

**Table 1:** Reliability of the PINSQ.

	Subscales	Cronbach′s alpha
**F1**	Professional self‐image (Q1‐Q6)	0.90
**F2**	Benefit of retention and risk of turnover (Q7‐Q10)	0.81
**F3**	Social comparison and self‐reflection (Q11‐Q13)	0.83
**F4**	Independence of career choice (Q14‐Q15)	0.42
**F5**	Social modelling (Q16‐Q17)	0.54
	**Professional Identity**	0.93

#### 2.5.3 Visual Analog Scale (VAS)

The VAS is a 100 mm line used to measure subjective experiences like pain. It helps reduce bias from individual interpretation of scales and is a valid and reliable tool widely used in healthcare research [54].

In COVID‐19‐related information, a set of five custom questions, all utilizing a VAS, was developed to assess specific aspects of the students′ pandemic experience, including their fear of COVID‐19, perceived informational support, perceived emotional support, impact of clinical training closure and impact of virtual education.

### 2.6 Data Analysis

Data were coded and analyzed using IBM SPSS Statistics for Windows version 27. Descriptive statistics including frequencies, percentages, means, standard deviations, minimums, and maximums, were used to summarize the sample characteristics and key variables.

For inferential analysis, the following tests were conducted: The Chi‐square (χ2) test was used to examine associations between categorical variables and different levels of professional identity. Pearson′s correlation coefficient (*r*) was used to assess the direction and strength of the linear relationship between continuous variables and the total professional identity score. A *p*‐value of less than 0.05 was considered statistically significant for all tests.

A linear regression analysis was performed to identify significant predictors of professional identity, using variables that showed significant associations in the initial Chi‐square and correlation tests. Multiple linear regression was conducted to identify factors associated with professional identity. The analysis was performed using the Enter method. Independent variables that were found to be significant in the univariate analysis were included in the regression model. An analysis of the model′s assumptions was conducted before interpretation. The normality of residuals was confirmed through a visual inspection of the P–P plot. The variance inflation factor (VIF) was used to assess multicollinearity, with all values falling between 1 and 3, indicating that the assumption of no multicollinearity was met. Furthermore, the Durbin–Watson test was used to check for the independence of errors; the resulting statistic of 2.31 confirmed that this assumption was also met. Given that all key assumptions were satisfied, the multiple linear regression model was deemed appropriate for the analysis.

## 3 Results

**Figure 1:**
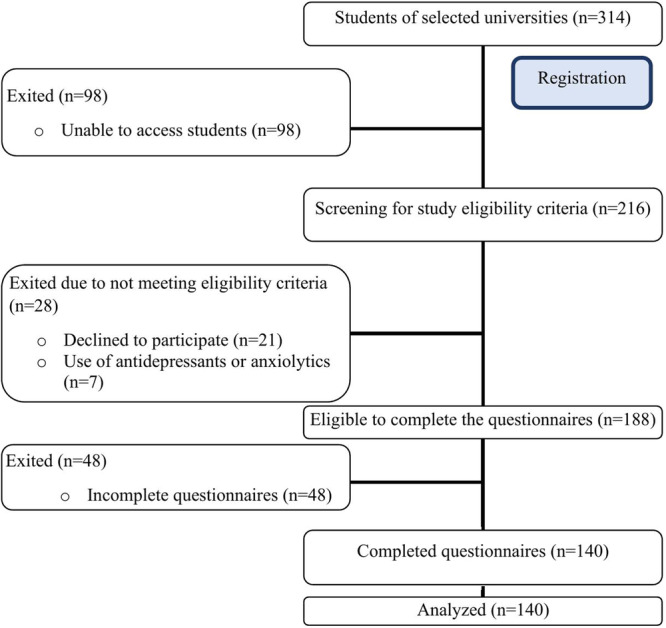
Workflow diagram of participant recruitment and selection.

Figure 1 presents a workflow diagram illustrating the process of participant recruitment and selection for the study.

The findings are detailed in the following subsections, which first outline the descriptive characteristics of the sample and then present the results of the inferential analyses.

### 3.1 Demographic characteristics

The demographic characteristics of the 140 participating midwifery students are detailed in Table 2. The average age of the participants was 25.11 years (SD = 3.15). The majority of students were single (67.1% [94/140]) and identified as Shi′a Muslim (85.7% [120/140]). The mean self‐reported monthly income was 6.27 (SD = 6.92) million Tomans.

**Table 2:** Characteristics of the participants.

Demographic characteristics		Frequency	Percentage
**Age (Year)**	Less than 25	94	67.1
	25–29	35	25.0
	30 and above	11	7.9
	Total	140	100
	Standard Deviation±Mean	11.15 ± 25.3	
	Minimum–Maximum	19–44	
**Religion**	Shi′a	120	85.7
	Sunni	20	14.3
	Total	140	100
**Marital status**	Married	46	32.9
	Single	94	67.1
	Total	140	100
**Monthly income (million Tomans)**	<5	76	54.3
	5–10	11	7.9
	10–15	52	37.1
	>15	1	0.7
	Total	140	100
	Standard Deviation±Mean	27.92 ± 6.6	
	Minimum–Maximum	0–30	
**Ethnicity**	Fars	37	26.4
	Kurd	5	3.6
	Turk	40	28.6
	Lur	7	5.0
	Gilak	14	10.0
	Other	37	26.4
	Total	140	100
**Place of residence**	Tehran	39	27.9
	Province	101	72.1
	Total	140	100
**Geographic region of residence**	Tehran	39	27.9
	North	42	30.0
	South	8	5.7
	East	12	8.6
	West	15	10.7
	Center	24	17.1
	Total	140	100
**Educational characteristics**			
**Are you the first university student in your family?**	Yes	63	45.0
	No	77	55.0
	Total	140	100
**Reason for choosing midwifery as a major**	My dream profession	19	13.6
	Parents′ insistence	5	3.6
	More job opportunities	65	46.4
	Other	51	36.4
	Total	140	100
**What number choice was midwifery for you as a major?**	First	14	10.0
	Second	36	25.7
	Third or more	90	64.3
	Total	140	100
**Current semester of study**	Graduate	58	41.4
	Year 2	6	4.3
	Year 3	22	15.7
	Year 4	54	38.6
	Total	140	100
**Do you want to be a midwife in the future?**	Yes	134	95.7
	No	6	4.3
	Total	140	100
**What was the impact of the COVID‐19 pandemic on your understanding of clinical midwifery work?**	No impact	7	5.0
	Clinical work is dangerous during a pandemic	32	22.9
	Clinical work and helping people during a pandemic are satisfying and honorable	101	72.1
	Total	140	100
**Socio‐Economic characteristics**			
**Father′s job**	Employee	92	65.7
	Self Employed	27	19.3
	Other	21	15.0
	Total	140	100
**Mother′s job**	Employee	61	43.6
	Self Employed	9	6.4
	Other	70	50.0
	Total	140	100
**Father′s education**	Diploma or less	26	18.6
	Bachelor′s degree	98	70.0
	Master′s degree and above	16	11.4
	Total	140	100
**Mother′s education**	Diploma or less	45	32.1
	Bachelor′s degree	91	65.0
	Master′s degree and above	4	2.9
	Total	140	100
**Father′s field of study**	Medical sciences	25	17.9
	Non‐ sciences	115	82.1
	Total	140	100
**Mother′s field of study**	Medical sciences	22	15.7
	Non‐Medical sciences	118	84.3
	Total	140	100
**Are you employed?**	Yes	68	48.6
	No	72	51.4
	Total	140	100
**If yes, is your job or previous work experience related to midwifery? ؟**	Unemployed	72	51.4
	Yes	62	44.3
	No	6	4.3
	Total	140	100
**Economic status**	Good	32	22.9
	Average	105	75.0
	poor	3	2.1
**Family′s monthly income (Million Toman)**	10–15	12	8.6
	15–20	31	22.1
	>20	97	69.3
	Total	140	100
	Standard Deviation±Mean	63.78 ± 30.13
	Minimum–Maximum	12–70
**Number of family members**	Two	41	29.3
	Three	21	15.0
	Four or more	78	55.7
	Total	140	100
**Possession of a personal laptop**	Yes	85	60.7
	No	55	39.3
	Total	140	100
**COVID‐19‐related information**			
**Have you ever contracted COVID‐19?**	Yes	110	78.6
	No	30	21.4
	Total	140	100
**Have you been hospitalized due to COVID‐19 infection?**	Yes	4	2.9
	No	136	97.1
	Total	140	100
**Has anyone in your family contracted COVID‐19?**	Yes	129	92.1
	No	11	7.9
	Total	140	100
**Has anyone in your family been hospitalized due to COVID‐19 infection?**	Yes	47	33.6
	No	93	66.4
	Total	140	100
**Has anyone in your family died due to COVID‐19 infection?**	Yes	10	7.1
	No	130	92.9
	Total	140	100
**How many doses of the COVID‐19 vaccine have you received so far?**	Single dose	1	.7
	Two doses	16	11.4
	Three doses	123	87.9
	Total	140	100
**Have all members of your family received three doses of the COVID‐19 vaccine?**	Yes	117	83.6
	No	23	16.4
	Total	140	100
**Have you volunteered with medical teams in hospitals during the COVID‐19 pandemic?**	Yes	58	41.4
	No	82	58.6
	Total	140	100
**If your answer to the above question is positive, how many hours of volunteer work have you done during the pandemic?**	Unemployed	82	58.6
	<20 h	11	7.9
	60–20 h	19	13.6
	>60 h	28	20.0
	Total	140	100
**In your opinion, what impact has the COVID pandemic had on the social image of medical professions?**	Positive impact	128	91.4
	Negative impact	7	5.0
	Not much impact	5	3.6
	Total	140	100
**What impact has the COVID‐19 pandemic had on your willingness to pursue a professional career in the future?**	Positive impact	108	77.1
	Negative impact	8	5.7
	Not much impact	24	17.1
	Total	140	100

Regarding ethnicity, the largest groups were Fars 26.4% [37/140] and other ethnicities 26.4% [37/140]. Most students resided in the province, 72.1% [101/140], with a notable portion originating from the northern regions of the country, 30% [42/140].

### 3.2 Educational Characteristics

Table 2 details the educational characteristics of the participating midwifery students. A notable proportion, 55% [77/140], were not first‐generation university students in their families.

Regarding their choice of major, the most frequently cited motivation was better job opportunities, reported by 46.4% [65/140] of participants. For the majority of students, 64.3% [90/140], midwifery was their third or higher choice of study. At the time of the survey, 41.4% [58/140] had already graduated. Despite the challenges, a majority (95.7% [134/140]) expressed their desire to pursue a career in midwifery. Furthermore, 72.1% [101/140] of students described their clinical work and the experience of assisting people during the COVID‐19 pandemic as satisfying and honorable.

### 3.3 Socio‐Economic characteristics

The socio‐economic characteristics of the participants are detailed in Table 2. Regarding parental background, a majority of fathers (65.7% [92/140]) were employed, while half of the mothers were homemakers. High levels of parental education were reported, with 70% [98/140] of fathers and 65% [91/140] of mothers holding a bachelor′s degree. The vast majority of parents had studied in non‐medical fields (82.1% [115/140] for fathers and 84.3% [118/140] for mothers).

In terms of the students′ own economic status, 51.4% [72/140] were unemployed. Among those with prior work experience, 44.3% [62/140] reported it was related to midwifery. Most students (75% [105/140]) described their economic situation as average, with a mean family income of 30.63 ± 13.78 million Tomans. Access to personal technology was common, with 60.7% [85/140] of students owning a personal laptop.

### 3.4 COVID‐19 Related Information

Tables 2 and 3 detail the COVID‐19‐related experiences of the midwifery students. Regarding personal health, 78.6% [110/140] of students reported having contracted COVID‐19, although the vast majority (97.1% [136/140]) had not been hospitalized due to the illness. The impact on students′ families was also significant, with 92.1% [129/140] reporting a family member had been infected; however, most of these cases (66.4% [93/140]) did not lead to hospitalization. A total of 7.1% [10/140] of students experienced the death of a family member due to the virus.

**Table 3:** Mean and standard deviation of COVID‐19 related information.

COVID‐19 related information	Mean	Standard deviation	Minimum	Maximum
**At the peak of the COVID‐19 pandemic, how much fear did you have of the coronavirus?**	7.34	1.845	1	10
**On a scale of 1 to 10, how would you evaluate the level of informational support you received from your professors during this period?**	**4.01**	**2.091**	1	10
**On a scale of 1 to 10, how would you evaluate the level of emotional support you received from your professors during this period?**	4.02	1.809	1	10
**What impact did the closure of clinical training have on your self‐confidence and feelings of professional efficacy?**	7.34	2.175	1	10
**What impact did virtual education have on your self‐confidence and feelings of professional efficacy?**	**7.44**	**1.719**	1	10

Vaccination rates were high, with 87.9% [123/140] of students and 83.6% [117/140] of their families having received three doses of the COVID‐19 vaccine. In terms of professional engagement, 41.4% [58/140] of students volunteered with medical teams in hospitals, with 20% [28/140] contributing over 60 h of service.

Perceptually, the pandemic′s influence was viewed positively in a professional context. A substantial majority of students, 91.4% [128/140], believed it improved the social image of medical professions, and 77.1% [108/140] felt it had a positive impact on their own future career aspirations.

### 3.5 Professional Identity

As shown in Table 4, the mean score for overall professional identity among midwifery students was 76.22 (SD = 9.44). Among the various dimensions of professional identity, “Independence in career choice” was reported with the lowest mean (M = 3.58, SD = 0.82), while “Social role modeling” received the highest mean score (M = 4.68, SD = 0.59).

**Table 4:** Mean and standard deviation of professional identity and its dimensions.

Professional identity and its dimensions	Minimum	Maximum	Mean	Standard deviation	Based on 5–1			
					Minimum	Maximum	Mean	Standard deviation
**Professional Self‐Image (6–30)**	14	30	27.62	3.80	2.33	5	4.60	0.63
**Benefit of retention and risk of turnover (4–20)**	8	20	18.04	2.96	2	5	4.51	0.73
**Social comparison and self‐reflection (3–15)**	6	15	14.02	1.75	2	5	4.67	0.58
**Independence of career choice (2–10)**	2	10	7.18	1.64	1	5	**3.58**	**0.82**
**Social modelling (2–10)**	5	10	9.36	1.19	2.5	5	**4.68**	**0.59**
**Professional identity (17–85)**	45	85	76.22	9.44	2.65	5	4.48	0.55

### 3.6 Relationships between Variables

The relationships between professional identity and various demographic, socioeconomic, educational, and pandemic‐related factors were examined, with key findings detailed in Tables 4, 5, 6, 7.

**Table 5:** Association Between Participant Characteristics and Professional Identity.

Demographic characteristics		Frequency	Professional identity		Test result
			Mean	Standard deviation	
**Religion**	Shi′a	120	75.79	9.71	– = 1.323 *df*=138† *p* = 0.188
	Sunni	20	78.80	7.28	
**Marital status**	Married	46	75.39	9.51	– = 0.727 *df*=138† *p* = 0.469
	Single	94	76.62	9.43	
**Ethnicity**	Fars	37	70.62	12.53	*F* = 5.905‡ ***P*** < **0.001**
	Kurd‐Lur	12	78.91	10.14	
	Turk	40	76.77	8.21	
	Gilak	14	76.64	8.39	
	Other	37	80.18	7.06	
**Place of residence**	Tehran	39	74.25	9.28	*t* = 1.538 *df*=138† *p* = 0.126
	County	101	76.98	8.02	
**Geographic region of residence**	Tehran	39	74.25	10.28	*F* = 2.001‡ *p* = 0.082
	North	42	75.85	7.87	
	South	8	75.75	8.14	
	East	12	78.5	7.7	
	West	15	72.8	9.25	
	Center	24	80.54	7.03	
**Age (Year)**					*r* = 0.421§ ***P*** < **0.001**
**Monthly income (million Tomans)**					*r* = 0.014§ *p* = 0.866
**Socio‐Economic characteristics**					
**Father′s job**	Employee	92	79.15	10.31	*F* = 16.112‡ ***P*** < **0.001**
	Self Employed	27	69.66	12.02	
	Other	21	71.81	11.50	
**Mother′s job**	Employee	61	77.45	8.05	*F* = 1.005‡ *p* = 0.369
	Self Employed	9	74.11	10.16	
	Other	70	75.41	10.41	
**Father′s education**	Diploma or less	26	67.11	11.69	*F* = 19.088‡ ***P*** < **0.00**
	Bachelor′s degree	98	78.57	9.96	
	Master′s degree and above	16	76.62	10.20	
**Mother′s education**	Diploma or less	45	72.64	10.73	*t* = 2.820 *df*=134† ***P*** = **0.006**
	Bachelor′s degree	91	77.80	9.44	
**Father′s field of study**	Medical sciences	25	79.96	6.46	*t* = 3.517 *df*=138† ***P*** = **0.001**
	Non‐ sciences	115	75.41	8.03	
**Mother′s field of study**	Medical sciences	22	76.35	10.44	*t* = 0.066 *df*=138† *p* = 0.948
	Non‐ sciences	118	76.20	9.41	
**Are you employed?**	Yes	68	76.48	10.101	*t* = 0.320 *df*=138† *p* = 0.749
	No	72	75.97	8.82	
**Economic status**	Good	32	74.50	10.80	*t* = 1.226 df=135† *p* = 0.223
	Average	105	76.81	8.89	
**Family′s monthly income (Million Toman)**	10‐15	12	70.58	9.36	*F* = 2.555‡ *p* = 0.081
	15‐20	31	77.58	8.04	
	More than 20	97	76.48	9.31	
**Number of family members**	Two	41	74.56	10.37	*F* = 1.479‡ *p* = 0.231
	Three	21	74.95	10.33	
	Four or More	78	77.43	9.25	
**Possession of a personal laptop**	Yes	85	74.34	10.77	*t* = 3.014 *df*=138† ***P*** = **0.001**
	No	55	79.12	8.89	
**Academic qualifications**					
**Are you the first university student in your family?**	Yes	63	76.25	9.82	*t* = 0.037 *df*=138† *p* = 0.971
	No	77	76.19	9.18	
**Reason for choosing midwifery as a major**	My ideal profession	19	75.21	8.21	*F* = 3.250‡ ***P*** = **0.042**
	More job opportunities	65	78.33	7.39	
	Other	56	74.11	10.37	
**What number choice was midwifery for you as a major?**	First	14	69.78	9.01	*F* = 3.999‡ ***P*** = **0.021**
	Second	36	76.05	10.05	
	Third or more	90	77.28	8.46	
**Current semester of study**	Graduate	58	77.91	9.94	*F* = 4.475‡ ***P*** = **0.005**
	Second year	6	69.33	12.98	
	Third year	22	71	11.53	
	Fourth year	54	77.29	8.62	
**What was the impact of the COVID‐19 pandemic on your understanding of clinical midwifery work?**	No impact	7	63	12.74	*F* = 14.942‡ ***P*** < **0.001**
	Clinical work is risky during an epidemic.	32	72.21	11.68	
	Clinical work and assisting people during a pandemic are satisfying and honorable.	101	78.40	9.92	
**COVID‐19‐related information**					
**Have you ever contracted COVID‐19?**	Yes	110	77.39	8.99	*t* = 2.227 *df*=138† ***P*** = **0.032**
	No	30	71.93	11.75	
**Have you been hospitalized due to COVID‐19 infection?**	Yes	129	76.09	9.46	*t* = 0.550 *df*=138† *p* = 0.583
	No	11	77.72	9.41	
**Has anyone in your family contracted COVID‐19?**	Yes	47	77.34	8.58	*t* = 0.997 *df*=138† *p* = 0.321
	No	93	75.65	9.84	
**Has anyone in your family been hospitalized due to COVID‐19 infection?**	Yes	10	72.60	12.37	*t* = 1.262 *df*=138† *p* = 0.209
	No	130	76.50	9.17	
**How many doses of the COVID‐19 vaccine have you received so far?**	Two doses	16	63.50	11.03	*t* = 4.748 *df*=137† ***P*** < **0.001**
	Three doses	123	78.11	8.21	
**Have all members of your family received three doses of the COVID‐19 vaccine?**	Yes	117	78.23	7.43	*t* = 4.760 *df*=138† ***P*** < **0.001**
	No	23	66	9.87	
**Have you volunteered with medical teams in hospitals during the COVID‐19 pandemic?**	Yes	58	75.84	10.68	*t* = 0.396 *df*=138† *p* = 0.693
	No	82	76.48	8.51	
**If your answer to the above question is positive, how many hours of volunteer work have you done during the pandemic?**	Unemployed	82	76.48	8.51	*F* = 0.455‡ *p* = 0.714
	Less than 20 h	11	75.27	12.69	
	60‐20 h	19	77.78	9.53	
	More than 60 h	28	74.75	10.35	
**In your opinion, what impact has the COVID pandemic had on the social image of medical professions?**	Positive impact	128	77.65	7.79	*F* = 24.176‡ ***P*** < **0.001**
	Negative impact	7	57.85	9.24	
	Not much impact	5	65.20	11.30	
**What impact has the COVID‐19 pandemic had on your willingness to pursue a professional career in the future?**	Positive impact	108	78.45	6.90	*F* = 22.999‡ ***P*** < **0.001**
	Negative impact	8	60.25	8.01	
	Not much impact	24	71.50	11.72	

^†^ *t* = Independent samples t‐test statistic;

^‡^ *F* = Analysis of Variance (ANOVA);

^§^ *r* = Pearson correlation coefficient.

**Table 6:** Correlation of professional identity with COVID‐19 related information in midwifery students of selected universities in the years 2016–2021.

COVID‐19‐related information	Professional identity
**At the peak of the COVID‐19 pandemic, how much fear did you have of the coronavirus?**	*r* = 0.336 ***p*** < **0.001** §
**On a scale of 1 to10, how would you evaluate the level of informational support you received from your professors during this period?**	*r* = −0.244 ***p*** = **0.004** §
**On a scale of 1 to10, how would you evaluate the level of emotional support you received from your professors during this period?**	*r* = −0.261 ***p*** = **0.002** §
**What impact did the closure of clinical training have on your self‐confidence and feelings of professional efficacy?**	*r* = 0.505 ***p*** < **0.001** §
**What impact did virtual education have on your self‐confidence and feelings of professional efficacy?**	*r* = 0.493 ***p*** < **0.001** §

^§^ *r* = Pearson correlation coefficient.

**Table 7:** Frequency distribution, mean, and standard deviation of professional identity by item in midwifery students of selected universities in the years 2016‐2021.

Dimensions of professional identity	Items		Strongly disagree (1)	Disagree (2)	Neutral (3)	Agree (4)	Strongly agree (5)	Mean (SD)
**Professional Self‐Image**	1	I like to be a midwife.	(2.1) 3	1 (0.7)	8 (5.7)	23 (16.4)	(75) 105	4.61 (0.81)
	2	I have no plan to change my career direction.	(0.7) 1	6 (4.3)	15 (10.7)	36 (25.7)	82 (58.6)	4.37 (0.89)
	3	I like my major and am positively preparing for my future work.	(0.7) 1	3 (2.1)	10 (7.1)	18 (12.9)	108 (77.1)	4.64 (0.77)
	4	I am proud of working in the field of midwifery.	0 (0)	2 (1.4)	7 (5)	18 (12.9)	113 (80.7)	4.73 (0.62)
	5	Being a midwife makes me happy.	3 (2.1)	1 (0.7)	10 (7.1)	15 (10.7)	111 (79.3)	4.64 (0.82)
	6	I am sure I will succeed in midwifery field.	1 (0.7)	1 (0.7)	9 (6.4)	27 (19.3)	102 (72.9)	4.63 (0.70)
**Benefit of retention and risk of turnover**	7	I like to communicate with predecessors of midwifery field.	2 (1.4)	6 (4.3)	11 (7.9)	15 (10.7)	106 (75.7)	4.55 (0.91)
	8	I will choose the job I like no matter what other people say.	1 (0.7)	4 (2.9)	4 (2.9)	25 (17.9)	106 (75.7)	4.65 (0.73)
	9	I have already devoted too much to midwifery career, including the economic aspect and effort, so I don′t want to leave the field of midwifery.	9 (6.4)	5 (3.6)	17 (12.1)	26 (18.6)	83 (59.3)	4.21 (1.18)
	10	I often explore my professional development by reflecting on my interests, personality and values in myself.	1 (0.7)	5 (3.6)	6 (4.3)	21 (15)	107 (76.4)	4.63 (0.79)
**Social comparison and self‐reflection**	11	Being a midwife make good use of my competence and advantage	0 (0)	4 (2.9)	9 (6.4)	19 (13.6)	108 (77.1)	4.65 (0.72)
	12	Turnover will cause me emotional wounds.	0 (0)	5 (3.6)	7 (5)	20 (14.3)	108 (77.1)	4.65 (0.73)
	13	My knowledge about the midwifery career mainly came from my parents, teacher and other relatives.	0 (0)	1 (0.7)	3 (2.1)	30 (21.4)	106 (75.7)	4.72 (0.53)
**Independence of career choice**	14	I try to know the condition of other career fields so that I can make my professional belief more strong.	(2.9) 4	(7.9) 11	(30) 42	(32.1) 45	(27.1) 38	3.73 (1.04)
	^15*^	Being a midwife can bring my creativity into play	(5.7) 8	(7.9) 11	(37.9) 53	(32.9) 46	(15.7) 22	**3.45 (1.03)**
**Social modelling**	16	Both one′s own ideal and surrounding factors should be considered during the process of career choice.	(2.1) 3	0 (0)	(6.4) 9	(15.7) 22	(75.7) 106	4.63 (0.79)
	17	I like to know more professional development stories of some successful persons in the midwifery field	(0.7) 1	(1.4) 2	(2.1) 3	(15) 21	(80.7) 113	**4.74 (0.64)**

### 3.7 Demographic and Socioeconomic Factors

Several demographic and socioeconomic variables were significantly associated with professional identity (Tables 4 and 5). Notably, ethnicity was a significant factor, with Fars students reporting lower mean scores than their Kurdish, Turkish, and other ethnic counterparts. A positive correlation was also observed between age and professional identity. Furthermore, professional identity was significantly associated with parental background, including the father′s occupation and education level, the mother′s education level, and the father′s field of study. Access to personal resources, such as owning a personal laptop, was also linked to a stronger professional identity.

### 3.8 Educational Influences

As shown in Table 5, educational characteristics were strongly related to professional identity. Students who cited “better job opportunities” as their motivation for choosing midwifery had higher identity scores. Conversely, and counter‐intuitively, students for whom midwifery was their first choice of study had lower scores than those who ranked it as a later choice. A significant difference was also found based on academic progression, with third‐year students reporting lower scores than fourth‐year students and graduates.

### 3.9 Pandemic‐Related Influences

The experience of the COVID‐19 pandemic also had a significant impact (Table 5). Students who had personally contracted COVID‐19 or had received three vaccine doses (along with their families) reported higher professional identity. A higher identity score was also associated with a positive perception of the pandemic′s impact on the social image of medical professions and on their own future career aspirations.

### 3.10 Correlations and Item‐Specific Findings

Further analysis revealed several significant correlations (Table 6). Professional identity was positively correlated with both the fear of contracting COVID‐19 and the perceived impact of clinical training closure on self‐confidence and professional efficacy. In contrast, a significant negative correlation was found between professional identity and the perceived levels of informational and emotional support received from professors.

An item‐level analysis (Tables 6 and 7) indicated that the highest level of agreement was for the statement, “I want to connect with midwifery specialists and senior individuals in this field” (mean = 4.74, SD = 0.64). The lowest agreement was for “My career choice was mainly made by my parents and those around me” (mean = 3.45, SD = 1.03), suggesting a strong sense of personal agency among the students.

### 3.11 Linear Regression

The multiple linear regression results, presented in Table 8, identified three significant predictors of professional identity. In the final model, age was a significant negative predictor (B = −0.979, β = −0.327, *p* < 0.001). For each 1‐year increase in age, the professional identity score was predicted to decrease by 0.979 points, holding other variables constant.

**Table 8:** Factors Associated with Professional Identity: Results of the Multiple Linear Regression Analysis.

Independent variables		B coefficient	Standard coefficient	95% Confidence interval (CI) for B min‐max	*t* Statistics	*p*‐value
**Age**		−0.979	−0.327	−1.469 to −0.489	−3.959	0.000
**Ethnicity**	**Kurd‐Lur**	3.564	0.106	−1.834 to 8.962	1.309	0.193
	**Turk**	3.187	0.153	−0.634 to 7.008	1.653	0.101
	**Gilak**	3.827	0.118	−1.227 to 8.881	1.501	0.136
	**Other**	4.192	0.193	0.026 to 8.358	1.995	0.049
	**Fars**	Reference category				
**Reason for choosing midwifery as a major**	**My dream profession**	2.658	0.095	−2.263 to 7.579	1.071	0.287
	**More job opportunities**	0.110	0.006	−3.073 to 3.292	0.068	0.946
	**Other**	Reference category				
**What number choice was midwifery for you as a major?**	**First**	−3.232	−0.103	–8.757 to 2.293	−1.160	0.249
	**Second**	1.316	0.061	−1.844 to 4.476	0.826	0.411
	**Third or more**	Reference category				
**Current semester of study**	**Year 2**	6.539	0.141	−1.201 to 14.280	1.675	0.097
	**Year 3**	−5.178	−0.201	−9.057 to −1.299	−2.646	0.009
	**Year 4**	−2.407	−0.123	−5.402 to 0.588	−1.593	0.114
	**Graduate**	Reference category				
**What was the impact of the COVID‐19 pandemic on your understanding of clinical midwifery work?**	**Clinical work during a pandemic is dangerous.**	–0.923	−0.041	−4.237 to 2.391	−0.552	0.582
	**Clinical work and helping people during a pandemic is satisfying and honorable.**	Reference category				
**Father′s job**	**Employee**	1.547	0.077	−2.690 to 5.784	0.724	0.471
	**Self Employed**	−0.035	−0.001	−4.594 to 4.525	−0.015	0.988
	**Other**	Reference category				
**Father′s education**	**Bachelor′s degree**	−1.295	−0.063	–6.317 to 3.727	−0.511	0.610
	**Master′s degree and above**	−3.502	−0.119	−9.998 to 2.994	−1.069	0.288
	**Diploma or less**	Reference category				
**Mother′s education**	**Bachelor′s degree**	1.887	0.093	−1.408 to 5.182	1.135	0.259
	**Diploma or less**	Reference category				
**Father′s field of study**	**Medical sciences**	0.732	0.029	−2.882 to 4.346	0.401	0.689
	**Non‐ sciences**	Reference category				
**Possession of a personal laptop**	**Yes**	−0.329	−0.017	−3.257 to 2.599	−0.223	0.824
	**No**	Reference category				
**Have you ever contracted COVID‐19?**	**Yes**	3.418	0.145	−0.077 to 6.914	1.939	0.055
	**No**	Reference category				
**How many doses of the COVID‐19 vaccine have you received so far?**	**Two doses**	−1.866	−0.063	−7.793 to 4.062	−0.624	0.534
	**Three doses**	Reference category				
**Have all members of your family received three doses of the COVID‐19 vaccine?**	**Yes**	2.263	0.089	−2.993 to 7.519	0.854	0.395
	**No**	Reference category				
**At the peak of the COVID‐19 pandemic, how much fear did you have of the coronavirus?**		0.746	0.146	−0.169 to 1.660	1.616	0.109
**On a scale of 1 to 10, how would you evaluate the level of informational support you received from your professors during this period?**		0.424	0.094	−0.659 to 1.508	0.776	0.439
**On a scale of 1 to 10, how would you evaluate the level of emotional support you received from your professors during this period?**		−0.514	−0.099	−1.776 to 0.748	−0.807	0.421
**What impact did the closure of clinical training have on your self‐confidence and feelings of professional efficacy?**		0.836	0.193	−0.134 to 1.805	1.708	0.091
**What impact did virtual education have on your self‐confidence and feelings of professional efficacy?**		0.247	0.045	−0.872 to 1.367	0.438	0.662

Furthermore, ethnicity was a significant factor. Compared to Fars students, students from other ethnic groups had a professional identity score that was, on average, 4.192 points higher (B = 4.192, β = 0.193, *p* = 0.049).

Finally, the academic year was a significant predictor. Compared to graduates, third‐year students had a professional identity score that was, on average, 5.178 points lower (B = −5.178, β = ‐0.201, *p* = 0.009).

The final prediction model for the professional identity score was as follows:

Professional Identity Score = 80.915 − 0.979(Age) + 4.192(Ethnicity Other) ‐ 5.178(Semester Year 3).

## 4 Discussion

This study explored the factors influencing professional identity among midwifery students in Tehran following the COVID‐19 pandemic. Our findings reveal that the pandemic had a complex and multifaceted impact, simultaneously disrupting clinical experiences and created significant stressors while reinforcing the societal value of the midwifery profession.

Consistent with previous research [55, 56, 57, 58], our findings underscore that professional identity formation is influenced by a variety of factors, including demographic characteristics, socioeconomic status, and educational experiences. Notably, students from certain ethnic minority groups and those with higher parental education levels reported a stronger professional identity. While our cross‐sectional design cannot establish causality, sociocultural theory [59] offers a possible explanation for our findings, particularly when considered within the Iranian social fabric. This theory suggests that individuals from minority backgrounds may build a stronger professional identity as a resilience mechanism to overcome systemic challenges and stereotypes [60, 61]. In a diverse country like Iran [62], students from different ethnic backgrounds might face unique social challenges in competitive university settings [63]. For these students, building a strong professional identity as a midwife is a powerful way to gain confidence and overcome potential prejudice [64, 65].

Similarly, students with highly educated parents often possess greater cultural capital, such as family support and a professional role model, which provides a stronger foundation for identity development [59, 66, 67, 68]. In the Iranian context, where medical careers are linked to family honor, support from educated parents is deeply cultural. This alignment with societal values provides a strong, culturally‐reinforced foundation for a student′s professional identity [69, 70]. Our finding that ethnicity impacts professional identity aligns with previous studies in healthcare student populations [71, 72], also in Malaysia [73], that highlight the significant role of social and cultural factors in in shaping how students perceive themselves and their future roles as midwives.

The pandemic presented a unique paradox for midwifery students. On one hand, the increased visibility and societal appreciation for healthcare workers appeared to bolster their professional identity. Our findings show that a positive perception of the pandemic′s impact on the social image of healthcare was associated with a stronger sense of professional identity. This suggests that witnessing the critical role of midwives during a crisis reinforced students′ pride and purpose in their chosen field.

On the other hand, the pandemic simultaneously created significant educational disruptions and personal stressors. The necessary shift to online learning and reduced access to clinical settings impacted students′ ability to engage in the hands‐on practice crucial for identity formation [74, 75]. Our results confirm the critical importance of clinical experience; students who managed to have more positive clinical exposures, despite the challenges, reported stronger professional identities. This underscores the urgent need for midwifery programs to develop resilient strategies to protect and prioritize students′ clinical learning, while also providing robust emotional and informational support to manage stressors related to personal safety and future uncertainty.

Our analysis also revealed two counterintuitive findings that challenge common assumptions. First, a negative correlation emerged between professional identity and perceived faculty support. We theorize this may be linked to student autonomy; students with a more developed professional identity likely hold higher expectations and may have been more critical of the remote support offered during the pandemic, perceiving it as insufficient. This suggests a potential mismatch between the support provided and the needs of highly engaged, autonomous students, a dynamic that merits further qualitative exploration.

Second, students who selected midwifery as their first choice exhibited lower mean professional identity scores than those for whom it was a later choice. This could be attributed to the phenomenon of reality shock [76]. First‐choice students may enter the field with idealized expectations that are quickly challenged by the demanding realities of the healthcare system, especially during a crisis. In contrast, those with more pragmatic motivations may possess a more resilient professional identity, less susceptible to such disillusionment.

### 4.1 Strengths and Limitations

This study provides valuable insights into a critical period for midwifery education, yet several limitations must be acknowledged. The most significant limitation is the low response rate (44.6%), which introduces a high risk of non‐response bias and severely limits the generalizability of our findings. It is plausible that students experiencing higher levels of burnout or a weaker professional identity were less motivated to participate, potentially leading to an overestimation of the average professional identity score in our sample. A potential limitation was the requirement of internet access for remote participants. However, given that university classes were held on mandatory virtual platforms during the pandemic, internet access was a prerequisite for all active students in the study population. Throughout the recruitment process, we did not encounter any eligible students who were unable to participate due to a lack of internet access. Consequently, while we acknowledge the theoretical potential for this bias, we believe it is unlikely to have significantly affected the composition of our final sample.

Furthermore, the cross‐sectional design limits any inference of causality. Methodological limitations also include the low internal consistency of two PINSQ subscales (“Independence of career choice”, α = 0.42 and “Social modelling”, α = 0.54), requiring that findings related to these dimensions be interpreted with caution. Finally, confirmatory factor analysis (CFA) was not performed to formally validate the instrument′s factor structure within this specific midwifery student population.

## 5 Conclusion

This study investigated the factors associated with professional identity among midwifery students in Tehran in the aftermath of the COVID‐19 pandemic. The findings highlight the complex interplay of demographic, socio‐economic, educational, and pandemic‐related experiences in shaping professional identity.

Notably, students from minority ethnic groups and those with higher parental education levels demonstrated stronger professional identity. Positive perceptions of the pandemic′s impact on the social image of healthcare professions and future career prospects were also associated with greater professional identity. The study underscores the importance of clinical experiences and the potential influence of the pandemic on students′ sense of professional efficacy and career aspirations. These findings have implications for midwifery education and practice. Midwifery programs should prioritize strategies to enhance students′ clinical experiences, provide emotional and informational support, and foster a sense of belonging within the professional community. Further research should explore the long‐term impact of the pandemic on the professional identity and career trajectories of midwifery students.

## Author Contributions

**Sahar Borji‐Navan:** conceptualization, methodology, investigation, project administration, validation, data curation, formal analysis, writing – original draft, writing – review and editing. **Homa Sadeghi Avval Shahr:** conceptualization, methodology, data curation, writing – review and editing, supervision, validation. **Shima Haghani:** conceptualization, methodology, validation, formal analysis, data curation, writing – review and editing. **Sanam Borji‐Navan:** conceptualization, formal analysis, writing – original draft, writing – review and editing.

## Funding

The authors received no specific funding for this work.

## Ethics Statement

The Ethics Committee of the Iran University of Medical Sciences approved our study protocol with the ethics code of IR. IUMS. REC.1401.878. We followed all the ethical principles of the World Medical Association Declaration of Helsinki for medical research involving human subjects. We obtained written informed consent from all study participants before their recruitment.

## Consent for Publication

The authors have nothing to report.

## Conflicts of Interest

The authors declare no conflicts of interest.

## Transparency Statement

The lead author, Homa Sadeghi, affirms that this manuscript is an honest, accurate, and transparent account of the study being reported; that no important aspects of the study have been omitted; and that any discrepancies from the study as planned (and, if relevant, registered) have been explained.

## Data Availability

The datasets used and/or analyzed during the present study are available from the corresponding author on reasonable request.
